# Eating Disorder Social Network Density: Its Impact on Diagnosis and Recovery

**DOI:** 10.1192/bjo.2025.10202

**Published:** 2025-06-20

**Authors:** A. Boisvert Jennifer, W. Andrew Harrell

**Affiliations:** ^1^Independent Practice, Beverly Hills, USA; ^2^University of Alberta, Edmonton, Canada

## Abstract

**Aims:** A handful of studies argue that ED treatment would benefit from a network analysis of social influences, particularly in girls and young women pressured by socio-culturally prescribed beauty standards, and reinforced by peers and family. The study’s aim was to investigate the impact of social network density on a person’s acquisition, perpetuation, and recovery from an eating disorder (ED). It was hypothesized that one’s connectedness within dense social networks of others with EDs would increase the likelihood of an ED diagnosis and resistance to treatment and recovery.

**Methods:** One thousand participants, largely from North America and Europe, completed an online survey of ED social networks. Respondents were asked whether they had an ED diagnosis, and if so, the diagnosis/typology, whether they knew others with an ED, whether they were in recovery, and, if so, the extent of social supports. Indices and latent structural equation model (SEM) variables were constructed from respondents’ identification of siblings, peers, friends, parents, other relatives, spouses, and neighbours with an ED. Similar indices were constructed for others identified as supportive of recovery. Social media influence was measured by asking if pro-anorexic or recovery websites were viewed. Data were analysed using bivariate statistics and Lavaan’s SEM *R* program.

**Results:** Social network density (knowing others with EDs) was highly predictive of ED diagnosis, including multiple EDs. Internet media was equally impactful. Same-sex siblings and peers had the greatest influence, exceeding parents or other relatives/friends. Networks of supportive others were highly predictive of recovery, outweighing negative ED models and media.

**Conclusion:** Our results were highly revealing of dense networks of family and peer models of EDs as well as supportive networks for recovery. The density/richness of social networks of others with an ED was highly predictive of an ED diagnosis, particularly of multiple EDs. Same-sex peers and siblings with an ED were especially strong influences. “Rich” day-to-day networks of multiple social contacts with EDs were associated with multiple ED diagnoses. Media appeared to complement these social contacts. However, only dense networks of supportive others were significantly predictive of recovery. Effective ED treatment requires a careful consideration of social influences who may model ED attitudes and behaviours; same-sex siblings and peers are especially critical. ED treatment and recovery might be compromised if these significant others model and reinforce a patient’s ED attitudes and behaviours.

